# Fitness cost of target-site and metabolic resistance to pyrethroids drives restoration of susceptibility in a highly resistant *Anopheles gambiae* population from Uganda

**DOI:** 10.1371/journal.pone.0271347

**Published:** 2022-07-26

**Authors:** Magellan Tchouakui, Ambrose Oruni, Tatiane Assatse, Claudine R. Manyaka, Micareme Tchoupo, Jonathan Kayondo, Charles S. Wondji

**Affiliations:** 1 Centre for Research in Infectious Diseases (CRID), Yaoundé, Cameroon; 2 Vector Biology Department, Liverpool School of Tropical Medicine, Liverpool, United Kingdom; 3 Entomology Department, Uganda Virus Research Institute (UVRI), Entebbe, Uganda; 4 Parasitology and Ecology Laboratory, Department of Animal Biology and Physiology, Faculty of Science, University of Yaoundé 1, Yaoundé, Cameroon; 5 International Institute of Tropical Agriculture (IITA), Yaoundé, Cameroon; Universidade Federal do Rio de Janeiro, BRAZIL

## Abstract

**Background:**

Insecticide resistance threatens the effectiveness of malaria vector control, calling for an urgent need to design suitable resistance management strategies. Here, we established the resistance profiling of an Ugandan *Anopheles gambiae* population to insecticides using WHO procedures and assessed the potential restoration of susceptibility in the hybrid line Mayuge/KISUMU in an insecticide-free environment for eighteen (18) generations.

**Results:**

This *An gambiae* population exhibited a very high intensity of resistance to permethrin, deltamethrin, and alphacypermethrin with a consistent loss of efficacy of all long-lasting insecticidal nets (LLINs) tested including PBO-based and new generation nets Interceptor G2 (IG2) and Royal guard. Molecular analysis revealed a fixation of the L1014S-kdr mutation together with the overexpression of some P450 metabolic genes (*CYP6Z1*, *CYP9K1*, *CYP6P1*, 3 & 4) besides the cuticular resistance-related genes (*CYP4G16*) and sensorial appendage proteins (*SAP1*, *SAP2*, and *SAP3*) but no *GSTe2* overexpression. In the absence of selection pressure, the mortality rate after exposure to insecticides increased significantly over generations, and restoration of susceptibility was observed for most of the insecticides in less than 10 generations. Accordingly, a significant reduction in the frequency of *KdrE* was observed after 13 generations coupled with reduced expression of most metabolic resistance genes.

**Conclusions:**

The results of this study show that the high intensity of pyrethroid resistance observed in *An gambiae* from Uganda associated with the loss of efficacy of LLINs could compromise vector control efforts. The study also highlights that an early rotation of insecticides could help manage resistance to insecticides by restoring the susceptibility. However, the persistence of *Kdr* mutation together with overexpression of some metabolic genes after many generations in the absence of selection pressure indicates the potential implication of modifiers alleviating the cost of resistance which needs to be further investigated.

## Introduction

Insecticide resistance is an increasing challenge for disease control [1]. Of particular concern is resistance to pyrethroid insecticides, given the high reliance on this class for malaria vector control. Populations of *Anopheles gambiae*, the main malaria vector, are exhibiting increasingly high levels of pyrethroid resistance, commonly measured by the knockdown resistance (*kdr*) mutations. This phenomenon leads to extensive loss of efficacy of LLINs including PBO-pyrethroid nets which is a growing problem in Uganda [2–5] and many other African countries [6–10].

Point mutations in the para-orthologous sodium channel gene disrupt insecticide binding to the voltage-gated sodium channels [11]. In this genomic region, two *kdr* mutations have been shown to be strongly associated with pyrethroid resistance in *Anopheles gambiae* including the Leucine to phenylalanine (L1014F-kdr west) and Leucine to Serine (L1014S-kdr east) mutations. Besides the *Kdr*, metabolic resistance is very common resistance mechanism in mosquitoes and considered to be more likely to cause control failure [12]. Compared to the well-characterised *kdr*, with available DNA-based diagnostic tools [13, 14], metabolic resistance still had only few molecular diagnostic tools preventing to assess the fitness cost associated with this resistance mechanism. This hampered the design of a suitable resistance management strategy to efficiently control the malaria vectors.

Polymorphism maintained at the *kdr* locus and metabolic genes in field populations of vectors at fine spatial scales despite strong insecticide selection pressure, is indicative of a fitness cost in the absence of insecticide [15, 16]. Although the availability of molecular marker for *kdr* helped to demonstrate evidence of such fitness costs in resistant mosquitoes [17–21], there is limited empirical evidence demonstrating restoration of pyrethroid susceptibility in insecticide-free environment in malaria vectors. Instead, studies tend to focus on selection towards resistance, describing increases in both *kdr* allele frequencies with pyrethroid exposure [15, 18, 22].

In this study, we extensively investigated the resistance profile of *An*. *gambiae* population from Mayuge (Eastern Uganda) and evaluated the fitness cost associated with the L1014S-kdr mutation in this population. Furthermore, we assessed potential restoration of susceptibility to the four recommended insecticide classes in the absence of selection pressure.

## Materials and methods

### Mosquito collection

Indoor resting and blood-fed female *Anopheles* mosquitoes were collected in Bubbalya (0°23′10.8′′N, 33°37′16.5′′E) in Mayuge (eastern Uganda) in February and October 2020. Mosquitoes were collected using electric aspirators morphologically identified as belonging to *An*. *funestus* group or *An*. *gambiae* s.l complex according to morphological keys [23]. These mosquitoes were kept in carton cups and fed with sugar until they became fully gravid prior to forced egg-laying in 1.5 ml microcentrifuge tubes and larvae reared to adults as previously described [24].

### Molecular identification of field-collected females

Oviposited and non oviposited females *An*. *gambiae* s.l were dissected into head plus thorax and abdomen for Genomic DNA (gDNA) extraction using the Livak method [25]. The SINE PCR assay [26] was used for the identification of the *An*. *gambiae* species.

### Insecticide susceptibility assays

The insecticide resistance profile of *An gambiae* s.l was assessed using the WHO tube bioassays [27]. After molecular identification, mosquitoes were split in two groups for bioassay including *An*. *gambiae* s.s., and *An*. *arabiensis*. Bioassay tests were performed with the pyrethroids type I (permethrin (0.75%)) and type II (deltamethrin and alphacypermethrin (0.05%)), the organochlorine DDT (4%), the carbamate bendiocarb (0.1%), and the organophosphate pyrimiphos-methyl (0.25%). Assays were performed at 25 ± 1° C and 70–80% relative humidity. For each test, four replicates of 20–25 F_1_ female mosquitoes, 2–5 day-old were exposed to insecticide-impregnated papers for 1h and final mortality recorded after a holding period of 24h. When resistance was observed with 1x (discriminant concentration (DC)) of pyrethroid (permethrin and deltamethrin), intensity bioassays were carried out with 5x DC and 10x DC of these insecticides. The intensity bioassays with 5x and 10x DC were performed following the WHO 2016 test procedure (WHO, 2016). Synergist assays with piperonyl butoxide (PBO; an inhibitor of cytochrome P450s) were performed for the potential involvement of P450’s genes.

### Insecticide-treated bed nets bioefficacy assays

Following the WHO guidelines for cone bioassays [28], the efficacy of the following LLINs including Olyset® Net (permethrin 2%) and Olyset® Plus net roof (permethrin 2% plus PBO 1% in the roof); PermaNet® 2.0 (deltamethrin 0.18%) and PermaNet® 3.0 side (deltamethrin 0.28%) was estimated using cone test approach. An untreated mosquito net was used as a control. Five replicates of ten F_1_ 2–5 days old females were placed in plastic cones enclosed with the mosquito net during 3 min exposure. Mosquitoes were then placed in small holding paper cups with cotton soaked in a 10% sugar solution. Mortality was determined 24 h later.

### Fitness cost study

#### Establishment of the mosquito strains

To facilitate the rearing of field mosquitoes, crossing was performed in February 2020 between *An gambiae* from Mayuge (MYG-R) and the susceptible laboratory KISUMU (KIS). The progeny (MYG/KIS) was intercrossed for several generations for resistance reversal study. To perform the crossing, pupae of each strain were collected and put individually in falcon tubes 15ml for individual emergence then the males of the resistant strain were mixed in the same cage with the females of the susceptible colony for random mating to generate the first generation as previously described [29]. At F_12_, the hybrid colony was backcrossed with the field strain to refresh the genetic background and the fitness cost associated with the L1014S-KdrE was evaluated using the F_3_ generation of the backcross.

#### Life trait experiments

All parameters were evaluated by simultaneously comparing fitness parameters (fecundity and fertility, larval mortality and adult longevity) between the mutant (1014S-RR), heterozygotes (L1014S-RS) and wild homozygote (L1014-SS), reared together in the same containers and under the same environmental conditions such as larval density and feeding, temperature and light exposition as done previously [29–31].

### Population cage experiments to assess a potential restoration of susceptibility

Cage experiments were conducted to assess a potential reversal to susceptibility. After crosses between male MYG-R and female *KISUMU*, the progeny obtained (MYG/KIS) were let in cages for intercrosses for eighteen (18) generations. In each generation, all mosquitoes irrespective of their genotypes were mixed in cages for intercrossing to generate the next generation. Each generation consisted in about 3 cages of at least 200 mosquitoes/cage of all genotypes. In the first generation, the frequency of the *KdrE_R* resistant allele was assessed and then monitored in following generations by genotyping a set of about 35 females aged between 2-5days old as well as expression on some candidate genes. Besides, bioassay was performed against permethrin 1x, 5x, 10x and 1x +PBO over generations (MYG/KIS F_2_, F_4_, F_7_ and F_13_) to confirm the restoration of susceptibility. Additional bioassays were performed at F_7_ and F_13_ for the four classes of insecticide commonly used and the susceptibility was compared to the field F1.

### Genotyping of resistance markers in *An*. *gambiae*

TaqMan assays with two labeled fluorochromes probes FAM and HEX were used to genotype the L1014F/L1014S-*kdr* (7), and the N1575Y mutation (35) associated with DDT and pyrethroid resistance in *An*. *gambiae* s.l. Also, the G119S-*ace*-*1* responsible for organophosphate and carbamate resistance in *An*. *gambiae* s.l. was also genotyped using TaqMan assays [32].

### Expression profile of resistance genes using real time quantitative PCR

To investigate the fitness cost associated with metabolic enzymes, the transcription profile of major insecticide resistance genes families including P450 metabolic genes (*CYP6Z1*, *CYP9K1*, *CYP6P1*, 3 & 4), GSTs (*GSTe2)*, cuticular resistance-related genes (*CYP4G16* and *CYP4G17*) and sensorial appendage proteins (*SAP1*, *SAP2*, and *SAP3*) overexpression were established in F3, F7, and F13 of the crossing compared to field F1 using *KISUMU* as susceptible reference strain. Total RNA from three biological replicates of 10 adults 2–5 days olds F1 for each group and similarly from *KISUMU* (susceptible lab strain) was extracted using Picopure RNA Isolation Kit (Arcturus). One microgram of RNA from each of the three biological replicates was used as a template for cDNA synthesis using the superscript III (Invitrogen) with oligo-dT20 and RNase H, following the manufacturer’s instructions.The relative expression level and fold-change (FC) was calculated individually according the 2^-ΔΔCT^ method [33] after normalisation with Ribosomal protein S7 (RSP7) and Elongation factor (EF).

#### Ethics statement

No permits were required for this work as the study only focused on mosquitoes with no involvement of human participants.

## Results

### Vector composition

In February, *An funestus* s.l was the predominant malaria vector (87.2%: 1636/1877) followed by *An gambiae* s.l. (12.8%: 241/1877) but in October, *An*. *gambiae* s.l was the main vector collected (80.1%: 1700/2121). Molecular identification of 100 *An*. *gambiae s*.*l* from Mayugue revealed that 78 (81.2%) were *An*. *gambiae* whereas the remaining (18.8%) were *An*. *arabiensis*.

### Insecticide susceptibility assays

**Fig 1:**
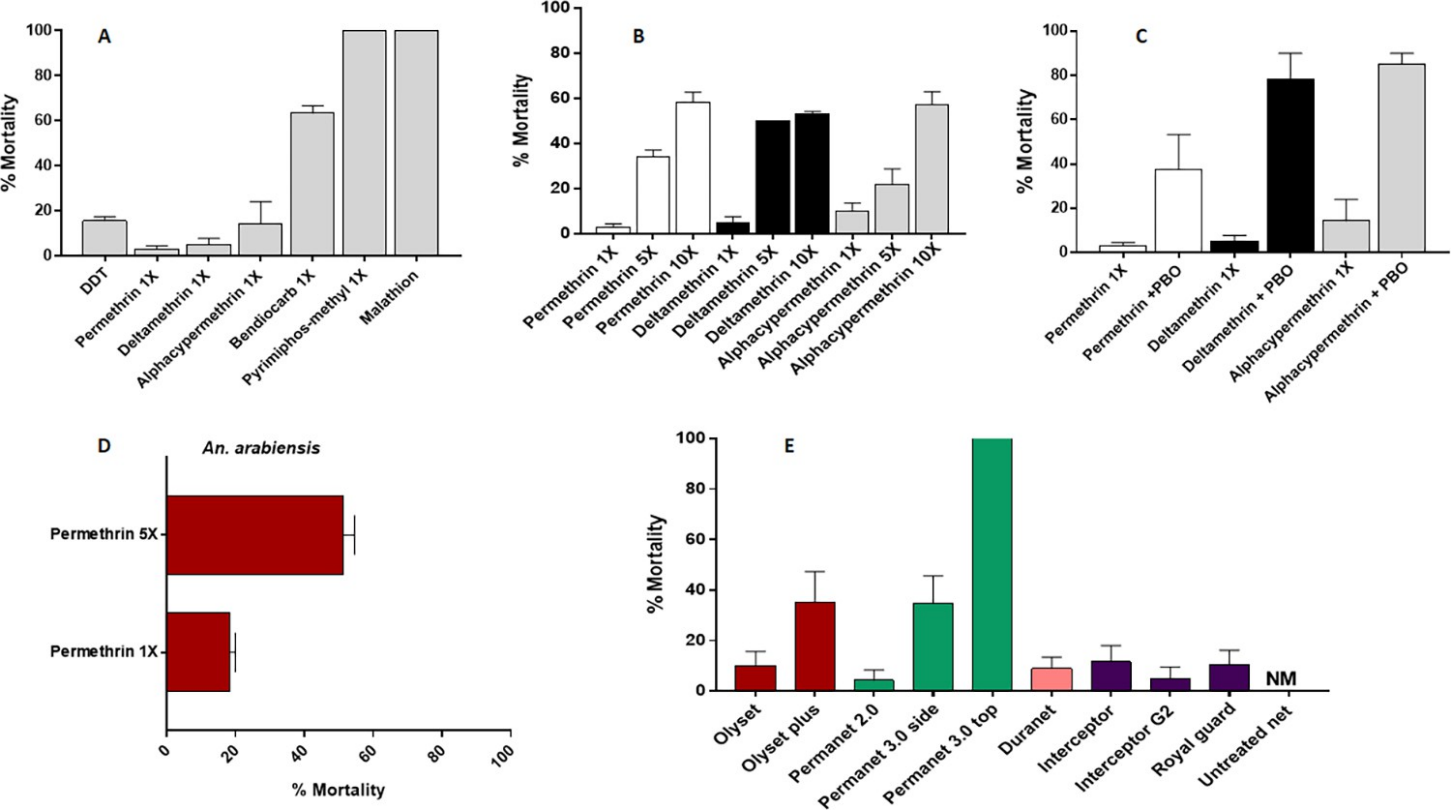
Susceptibility profile of *An*. *gambiae s*.*l* population from Mayuge. A) susceptibility profile of females *An*. *gambiae*; B) resistance intensity with 5× and 10× the diagnostic concentrations of permethrin and deltamethrin and Alphacypermethrin C) effect of pre-exposure to synergist PBO against pyrethroids. D) Susceptibility profile and intensity of females *An*. *arabiensis* and E) bio-efficacy of different commercial LLINs against *An*. *gambiae*. Results are average of percentage mortalities ± SEM; Results are average of percentage mortalities from four replicates each ± SEM.

F_1_ progeny from field-collected females *An gambiae* in October showed extremely high resistance to permethrin, deltamethrin and alphacypermethrin. F_1_ females from Mayuge showed 1.52 ± 1.52%, 3.19 ± 1.96% and 0% mortality 24h after exposure to permethrin 1x, deltamethrin 1x and alphacypermethrin 1x respectively **(Fig 1A)**. This *An*. *gambiae* population was also resistant to carbamate bendiocarb 1x, with mortality rate of 70.21 ± 1.79% **(Fig 1A)**. High resistance was noted against the organochlorine, DDT (mortality = 15.51 ± 1.88% **(Fig 1A)**. A full susceptibility was observed with the organophosphate, pyrimiphos-methyl 1x with a 100% mortality rate. Due to low sample size, *An arabiensis* was tested only to permethrin exhibiting a mortality of 18.33 ± 1.67%.

This population exhibited a mortality rate of 26.3 ± 3.4% and 91.12 ± 2.2% to permethrin 5x and 10x respectively **(Fig 1B)** showing a high intensity of resistance to permethrin. The mortality of 45.8 ± 4.2% and 70.5 ± 5.8% was observed after exposure to deltamethrin 5x and 10x respectively indicating a high intensity of resistance to deltamethrin in Mayuge **(Fig 1B)**. Similar observations were made for alphacypermethrin with mortality rates of 13.6 ± 4.1% and 59.6 ± 1.3% for 5x and 10x **(Fig 1B)**. *An*. *arabiensis* also displayed a high intensity of resistance to permethrin 5x with mortality of 51.3 ± 3.3% **(Fig 1D)**. Synergist assays performed with PBO revealed partial recovery of susceptibility after exposure to permethrin (from 1.52 ± 1.52% to 37.4 ± 15.9% mortality) and greater recovery with deltamethrin and alphacypermethrin with mortality rate of 78.33 ± 11.7% and 85.0 ± 5.0% respectively **(Fig 1C)**.

### Bioefficacy of insecticide-treated bed nets

Standard nets (Olyset and PermaNet 2.0, DuraNet and Interceptor) showed very low efficacy against this population of malaria vector with mortality rate of less than 15% for all these nets **(Fig 1E)**. However, the PBO-based net PermaNet 3.0 showed optimal efficacy with 100% mortality observed **(Fig 1E)**. In contrast, the Olyset plus (PBO-based net) showed very low efficacy (mortality rate = 35.12 ± 12.1%) confirming the very low mortality recorded with Permethrin + PBO in this site. New generation nets (NGN) (Interceptor G2 and Royal Guard) which combine a pyrethroid and non-pyrethroid insecticides induced very low mortality against this population **(Fig 1E)**. However, beside the direct killing effect, NGN such as Royal acts by sterilising the female which could help reduce the vector density.

### Distribution of insecticide resistance markers in field collected *An*. *gambiae*

The L1014F-KdrW mutation was completely absent in both *An*. *gambiae* and *An*. *arabiensis* from Mayuge. However, the L1014S-KdrE mutation was fixed in *An gambiae* (100% RR) and completely absent in *An*. *arabiensis*. The N1575Y-kdr mutation conferring pyrethroid resistance and the G119S-Ace1 mutation conferring carbamate resistance were completely absent in both species.

### Fitness cost associated with the L1014S mutation

**Fig 2:**
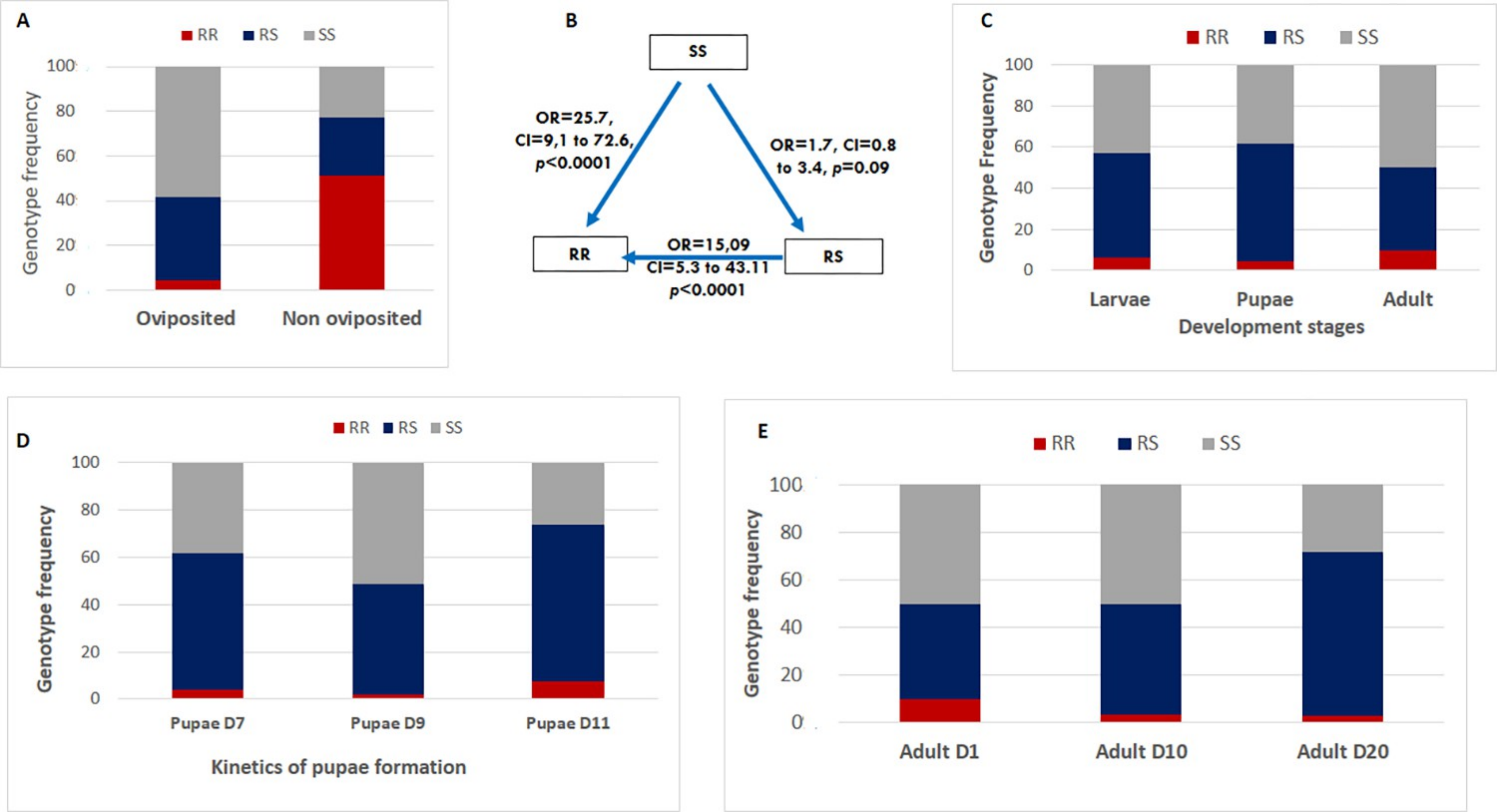
Influence of of the L1014S-KdrE on key life traits of *An*. *gambiae*. (A) and (B) Schematic representation of the impact of L1014S genotypes on egg-laying success with odd ratio (OR); (C) Distribution of the L1014S genotypes at different developmental stages of the hybrid MYG/KIS; D) the proportion of pupae obtained in D7, D9 and D11 of development; E) influence of L1014S on the adult longevity of *An*. *gambiae*.

In the hybrid strain MYG/KIS, mosquitoes with wild allele (L1014) displayed significant greater ability to lay eggs in the second gonotrophic cycle compared to those with the 1014S mutant allele (χ^2^ = 54.8; P<0.0001). However, no difference was found in the number of eggs laid by mosquitoes with mutant genotype (RR) (Mean = 77) compared to heterozygote RS (Mean = 64±6.3) and those with wild genotype (SS) (Mean = 55±6.4) as well as the hatch rate probably due to very low samples size of mosquitoes with RR genotype (Fig 2A). Assessment of the odds ratio (OR) showed that the ability of SS mosquitoes to lay eggs was higher compared to RR (OR = 25.7; confidence interval (CI) 95%: 9.1–72.6; P < 0.0001) but suggesting an association between the L1014S mutation and reduced fecundity although no significant difference was observed when compared to RS (OR = 1.7; CI 95%: 0.8–3.4; p = 0.09). Heterozygote also showed a higher ability to lay eggs compared to RR (OR = 15.09; CI 95%: 5.3–43.1; P < 0.0001) **(Fig 2B)** confirming the burden of the 1014S allele on the female fecundity.

No difference was observed concerning the mortality rate from the larvae to the adult for the three genotypes **(Fig 2C)**. Assessment of the rate of pupae formation by comparing the frequency of the genotypes of the pupae obtained in D7, D9 and D11 showed that mosquitoes with the wild genotype developed significantly faster than homozygote mutants and heterozygote mosquitoes as their frequency decreased significantly from D7 (38%) and D9 (51%) to D11 (26%) (χ^2^ = 4.4; p = 0.03) when that of RS and RR were increasing indicating a fitness cost of the resistant allele **(Fig 2B)**. Genotyping of thirty (30) live mosquitoes at D1, D10, and D20 post emergence to assess the association between the L1014S mutation and adult longevity revealed an heterozygote advantage. Comparison of genotypes frequency showed a decrease proportion of mosquitoes with both SS and RR genotypes from D1 to D20 (χ2 = 21.2; p = 0.0017) **(Fig 2B and 2D)** compared to heterozygote indicating that heterozygote mosquitoes live longer than those with homozygote genotypes.

### Restoration of susceptibility in the hybrid strain MYG/KIS

**Fig 3:**
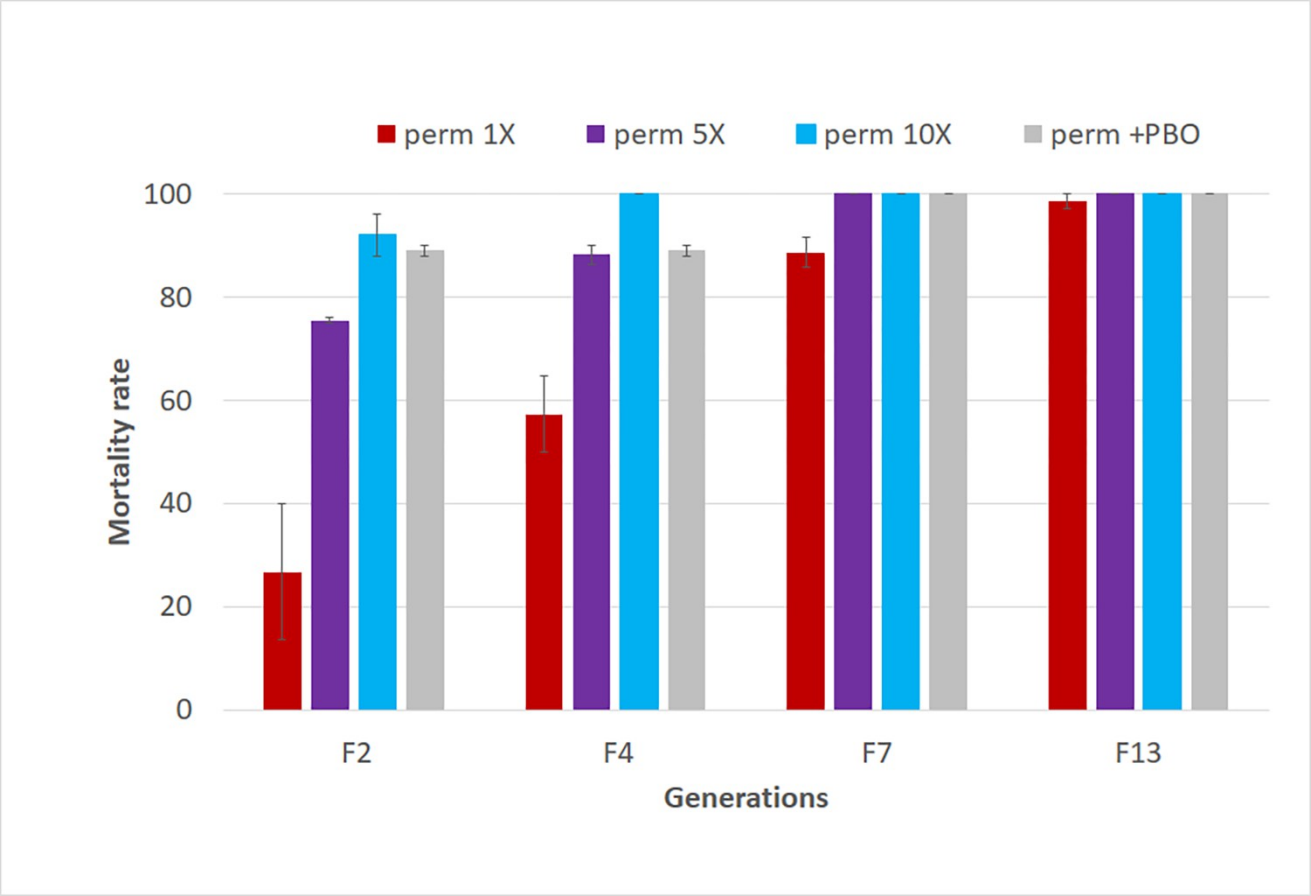
Susceptibility profile of the hybrid MYG/KIS to permethrin over 13 generation in the absence of selection. Results of WHO tube bioassays with permethrin 1x, 5x and 10x and PBO+ permethrin 1x. Results are average of percentage mortalities ± SEM of four replicates.

At F_2_ generation, the hybrid strain *MYG/KIS* exhibited a mortality rate of 26.82 ± 13.18%, 75.50 ± 0.5% and 89.0 ± 1.0% to permethrin 1x, 5x and 10x respectively **(Fig 3).** Synergist test performed at this generation revealed that P450 monoxygenases are playing a major role in the resistance with a significant recovery of susceptibility (mortality rate = 92 ± 4%) after PBO exposure.

**Fig 4:**
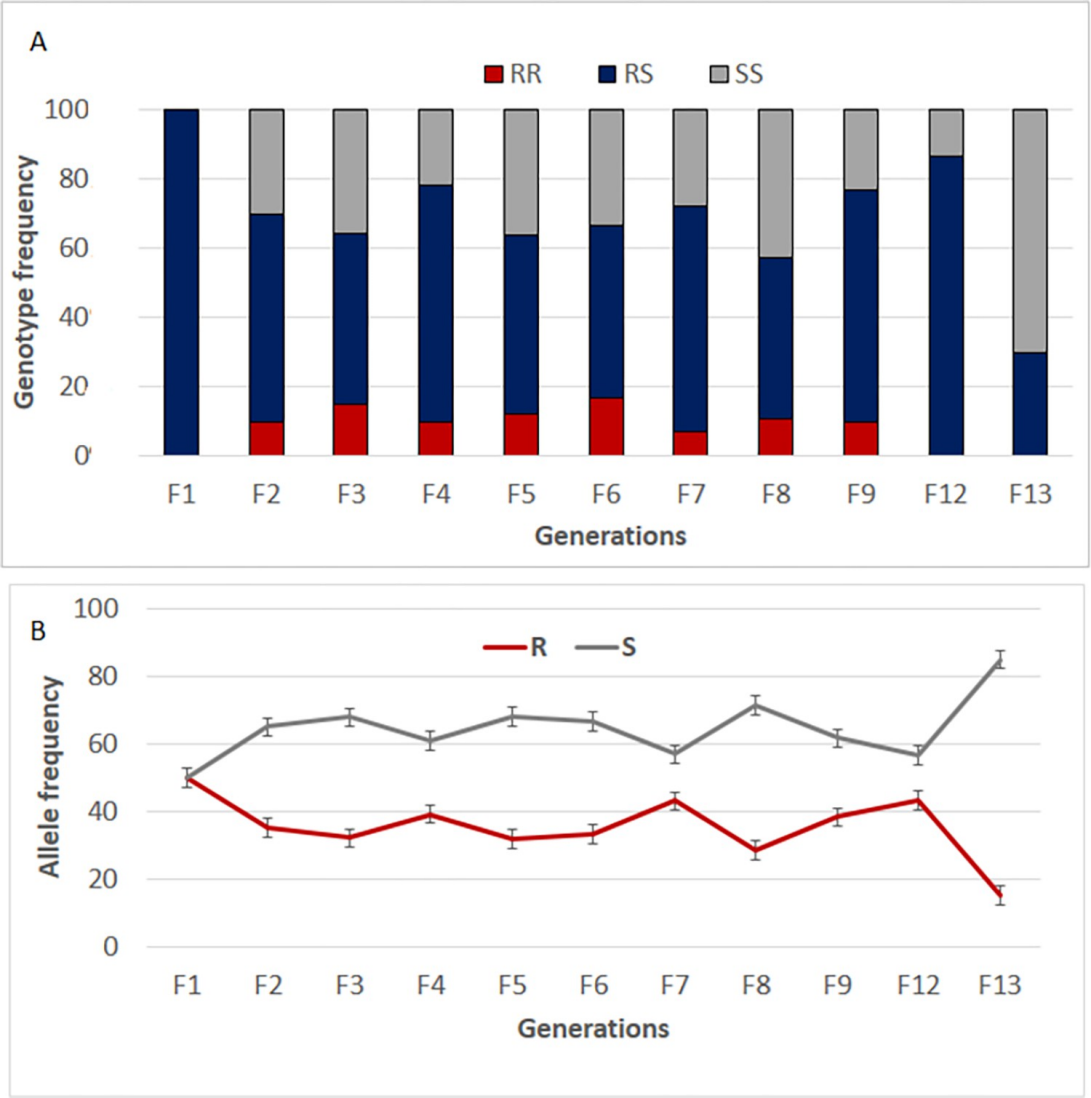
Evaluation of the reversal to susceptibility in the hybrid colony MYG/KIS. Changes in the L1014S genotypes (A) and allele (B) over13 generations in the insecticides free-environment; F represents each generation.

Genotyping of L1014F mutation across generations revealed that the frequency of 50% of the 1014S mutant allele obtained in F_1_ generation of the crossing decreased significantly in the next generations **(Fig 4A & 4B)**. A significant and consistent increase in the proportion of wild and heterozygote mosquitoes was observed from F_2_ to F_13_ moving from 50% to 15% (χ2 = 9.6; p = 0.002) with a predominance of heterozygotes indicating a fitness cost associated with the 1014S allele which lead to reversal to susceptibility **(Fig 4A & 4B)**. Accordingly the deviation to Hardy-Weinberg equilibrium was noticed in F4 (χ^2^ = 8; P < 0.001), F9 (χ^2^ = 3.8; P < 0.05), and F10 (χ^2^ = 17.5; P < 0.0001) confirming the fitness cost associated with the *kdr* mutation. However, the equilibrium was restored in some generation including F5-F9 and F12 to above (P>0.05) highlighting the implication of genetic modifiers alleviating the cost associated with the 1014S-kdr resistant allele. Nevertheless, restoration of susceptibility was observed after bioassay performed with permethrin over generations **(Fig 3)**.

**Fig 5:**
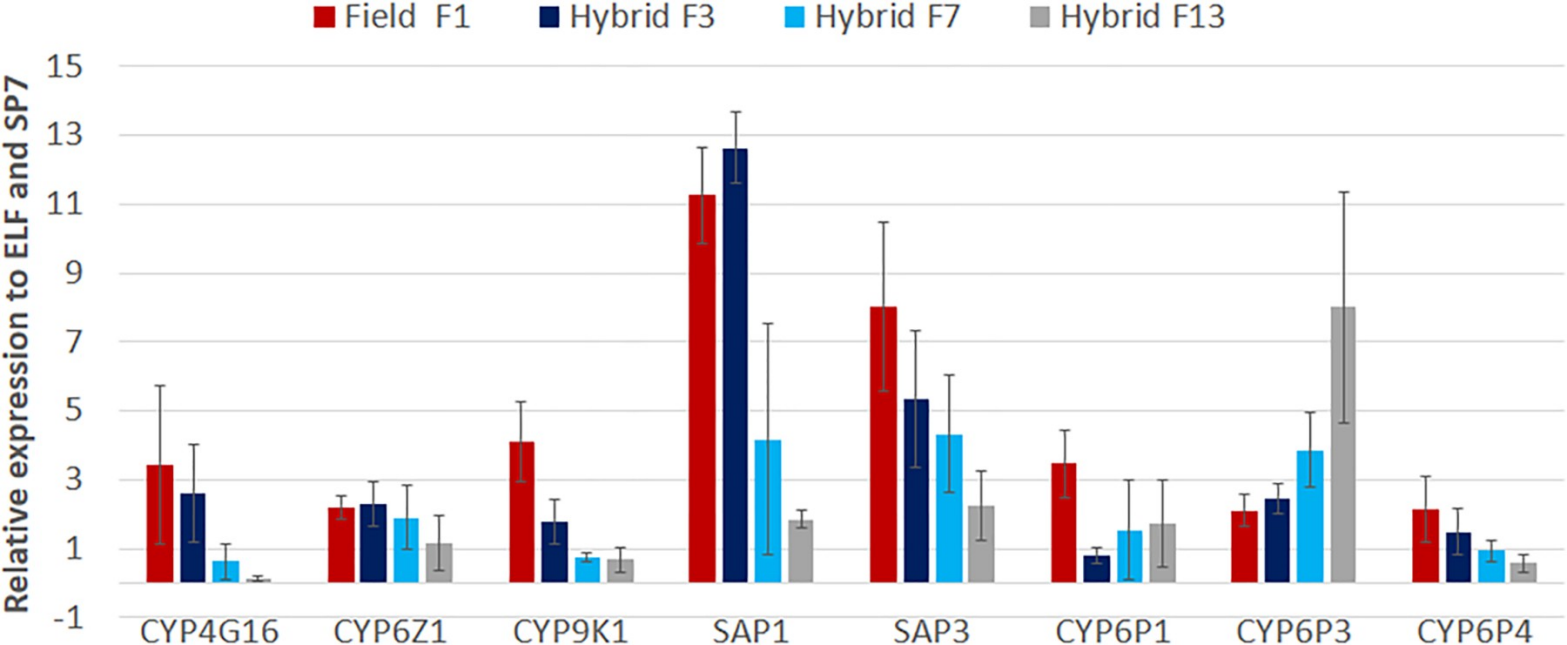
Differential expression by quantitative reverse-transcription polymerase chain reaction of key metabolic genes in the hybrid MYG/KIS over generation compared to field F1 unexposed. Histograms represent the fold-change of the genes in the hybrid MYG/KIS mosquitoes across generations relative to the pyrethroid-susceptible KISUMU laboratory strain.

For metabolic genes, qPCR results revealed a significant overexpression of several P450 resistant genes (*CYP6Z1 (2*.*2*± 0.3), *CYP9K1 (4*.*1*± 1.1), *CYP6P1 (3*.*5* ± 1.0), *CYP6P3(2*.*1*± 0.5) & *CYP6P4(2*.*1*± 0.9)) besides the cuticular genes (*CYP4G16 (3*.*4*± 2.3)) and sensorial appendage proteins (*SAP1(11*.*2*± 1.4), *SAP2(2*.*9* ± 1.2), and *SAP3(8*.*01*± 2.5)) with no overexpression of *GSTe2 (0*.*5*± 0.1) **(Table 1)**. The level of expression of these genes decreased significantly over generations (from F3-F13) (except for *CYP6P3*) suggesting a recovery of susceptibility as observed with the kdr-E marker **(Fig 5)**. The expression of decreased by 3.3, 1.1, 3.1, 9.4, 6.9, 1.7 and 1.52 folds for *CYP4G16*, *CYP6Z1*, *CYP9K1*, *SAP1*, *SAP3*, *CYP6P1* and *CYP6P3* respectively.

**Table 1:** Relative expression of metabolic resistance gene in F1 *An gambiae* from Mayuge.

Genes	Fold-change	SEM
***CYP4G16***	3.42	2,30
***CYP4G17***	1.62	1,32
***CYP6M2***	0,78	0,36
***CYP6Z1***	2,21	0,33
***CYP6Z2***	0,42	0,08
***CYP9K1***	4,11	1,15
***GSTe2***	0,45	0,16
***SAP1***	11,25	1,40
***SAP2***	2,91	1,18
***SAP3***	8,01	2,46
***CYP6P1***	3,46	0,99
***CYP6P3***	2,10	0,48
***CYP6P4***	2,14	0,94

**Fig 6:**
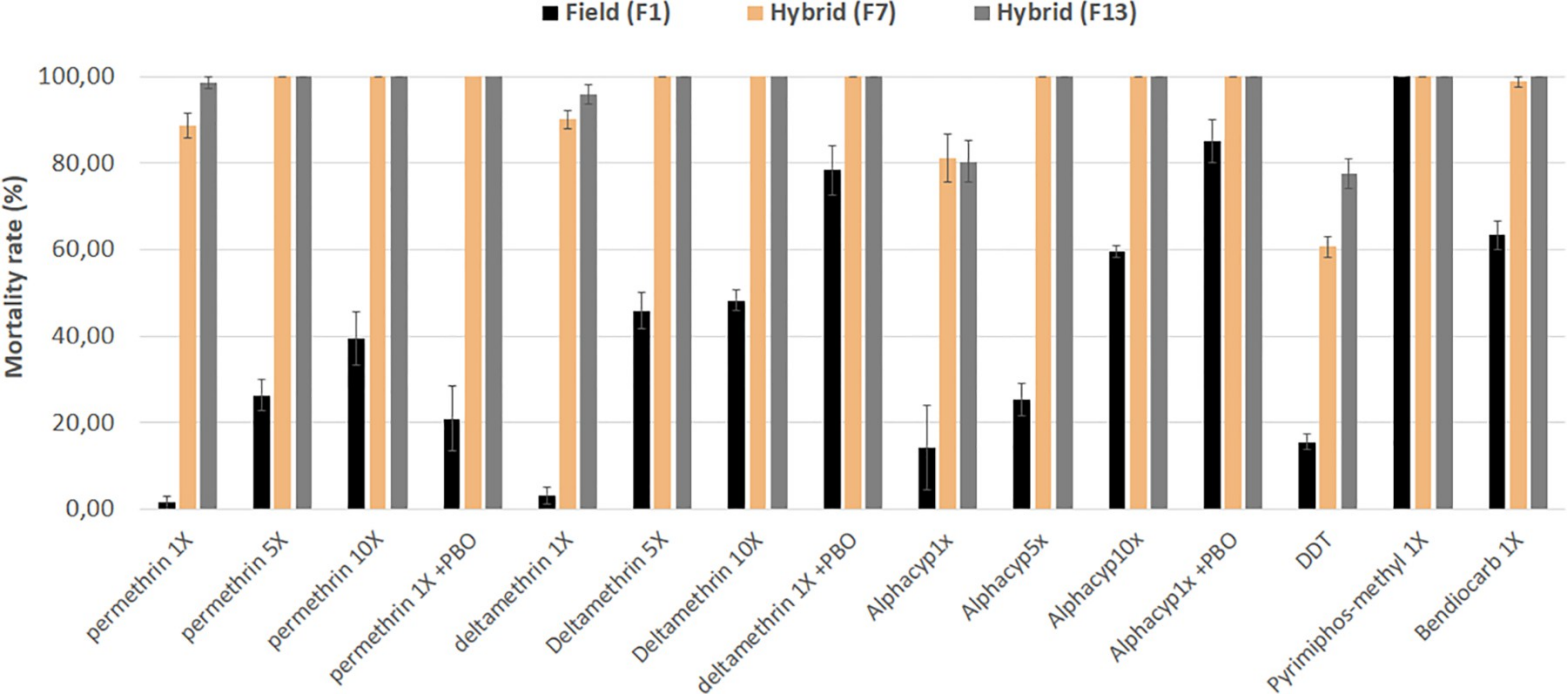
Evaluation of the restoration of susceptibility in the hybrid colony MYG/KIS. Determination of susceptibility to all the four classes of insecticides, resistance intensity with 5× and 10× the diagnostic concentrations of pyrethroids and effect of pre-exposure to synergist PBO against pyrethroids type I and type II in comparison with field F0. Results are average of percentage mortalities from four replicates each ± SEM.

After seven generations in insecticide-free environment (F_7_), the hybrid strain became susceptible to most of the insecticides tested including pyrethroids, carbamates and pyrimiphos methyl **(Fig 6)**. A full susceptibility to all the insecticides tested was noticed at the 13^th^ generation except for DDT and alphacypermethrin where a mortality rate of 77.60% and 80.40% was observed **(Fig 6)** indicating potential implication of genetic modifiers alleviating the cost of resistance to these insecticides and showing that reversal for these insecticides needs more time. For this reason, the strain was maintained in the insectary until F_18_ where a mortality rate of 93.1% was noted for alphacypermethrin and 90.2% for DDT.

## Discussion

In this study, we extensively investigated the resistance profile of *An*. *gambiae* population from Mayuge (Eastern Uganda) and evaluated the fitness cost associated with the *kdr* mutation and metabolic genes in this population. Furthermore, we assessed potential restoration of susceptibility to the four recommended insecticide classes in the absence of selection pressure.

### Elevated resistance intensity in the *An gambiae* population from Uganda

The Ugandan *An*. *gambiae* in this study exhibited extremely high level of resistance to pyrethroid with drastical loss of efficacy of insecticidal treated LLINs including new generation nets. A similar high level of resistance was reported recently in *An*. *funestus* s.s. populations from for the same location [34]. Such high intensity with loss in the efficacy of standard and PBO-based nets observed in *An gambiae* from Mayuge is similar to the observations of Okia *et al*. (2018) in Tororo a location in Eastern Uganda. The escalation of pyrethroid and DDT resistance observed in the Mayuge *An*. *gambiae* population is higher than resistance reported in other locations such as Kome, southern Chad (permethrin, 26.7% mortality, deltamethrin, 25.4% and DDT, 41.7%) [10]; and in Auyo, northern Nigeria (deltamethrin, 78.4% mortality and DDT, 44%); and in Djenne [35]. This increase may be associated with insecticide selective pressure imposed by the massive use of LLINs in Uganda since 2006 combined with agriculture. The same level of resistance was reported in An. *coluzzii* Cameroon and Chad [10], *An*. *funestus* in Cameroon [7] for which no mortality was noticed after exposure to the synergist net Olyset Plus. This was reported also a few years ago in Southern Mozambique where a complete loss of the efficacy of Olyset® Net, and PermaNet® 2.0, the two most distributed LLINs across Africa [8, 36], was noticed. Resistance was noted also for the carbamate bendiocarb althought the Ace1 mutation was absent in this population showing that such insecticide could not be an alternative to pyrethroid for IRS. This justifies the deployment of the new generation net Royal guard in the location which acts by sterilizing the female mosquitoes. The full susceptibility to the organophosphate pyrimiphos-methyl, seeing in this location suggests that this insecticide class is the most suitable for IRS against *An gambiae* and supports the use of Actellic for IRS in many districts of the country.

Genotyping of resistant markers revealed a fixation of the L1014S-kdr mutation showing that this mechanism is responsible in majority for pyrethroids resistance in these *An gambiae* mosquitoes particularly to permethrin for which a lower recovery of susceptibility was obtained after PBO pre-exposure. The same synergist assay with PBO helped to significantly recover the susceptibility to deltamethrin and alphacypermethrin showing that many P450s are involved in the extremely high intensity of resistance observed to type II pyrethroid in this location. qPCR analysis on the F*1* progeny from field collected females revealed the overexpression of some P450 metabolic genes (*CYP6Z1*, *CYP9K1*, *CYP6P1*,3&4) besides the cuticular genes (*CYP4G16*) and sensorial appendage proteins (*SAP1*, *SAP2*, and *SAP3*) with almost no overexpression of *GSTe2*. This indicates that the 1014S resistant allele combines with P450, cuticular and sensorial genes to accelerate the intensity of resistance in this location.

### Restoration of susceptibility in the resistant *An*. *gambiae* hybrid line

For successful insecticide resistance management, regular monitoring of resistance development and underlying mechanisms are key. Among the insecticide-resistant management strategies, rotational and mixture of insecticides to retard or reverse the spread of resistance are the most efficient and they are mainly based on the assumption of resistance having a fitness cost in the absence of selection. Knowledge of the reversal rate for insecticides such as pyrethroids is therefore crucial before implementing any resistance management strategies in the field based on rotation/mixture of insecticides.

In this study, the hybrid strain MYG/KIS raised in the absence of insecticide selection pressure became progressively more susceptible compared to the parental population. This suggests that resistance under field conditions can diminish after few generations without insecticide pressure. This implies a higher fitness of the susceptible phenotypes relative to resistant phenotypes in the untreated environment as observed for the *kdr* on some life traits such as fecundity, larval development and longevity. Some cases of reversal of insecticide resistance of culicines (*Aedes aegypti* and *Culex pipiens*) and *Anopheles gambiae* raised in the absence of insecticide in semi-field and laboratory conditions have been reported [15, 16, 37, 38]. The same reversal has been observed in pests such as cotton bollworm (*Helicoverpa armigera*) in Benin West Africa [39]. In this species, when the use the level of resistance increased quickly during the application of insecticides and decreased when treatment was suspended in the field. On the other hand, it was reported an increasing phenotypic resistance coupled to increase frequency of *kdr* resistant alleles and over-expression of monoxygenases/esterase in *An*. *gambiae* subjected to deltamethrin selection pressure in the lab [15, 16] indicating the rapid development of resistance in the environment undergoing continues insecticide use.

The frequency and the degree of dominance of resistant genes in a population is one of the factors able to influence the intensity and development of resistance in a population [40]. In this study, the frequency of 1014S mutant allele decreased consistently over generations from F_2_ to F_13_ indicating the importance of *kdr* marker in monitoring insecticide resistance development in an environment experiencing intensive use of insecticides. These results agree with the assumption that observed reversal of insecticide resistance in the absence of insecticide pressure is associated to fitness costs as shown previously [41]. Accordingly, a high fitness cost was observed in mosquitoes harbouring the resistant allele for the *kdr* marker although a heterozygote advantage was observed for some traits. Such heterozygote advantage was previously reported for the mating competitiveness in *An gambiae* for the *kdr* and *Rdl* target site resistance [42]. This could help maintaining the resistant allele in the population which could be rapidly re-selected if the selection pressure increases. Because of the lack of molecular markers for metabolic resistance in *An gambiae*, it was not possible to study the fitness cost associated with this mechanism as done previously for GSTs and P450s in *An*. *funestus* [29–31]. Nevertheless, we observed that the expression level of monooxygenases, cuticular and sensorial appendage proteins decreased significantly over generations in this hybrid strain maintained without insecticide pressure, indicating that metabolic resistance decreased in the absence of insecticide selection pressure likely as a consequence of related fitness cost.

Furthermore, we observed that when the hybrid resistant strain was pre-exposed to PBO before exposure to permethrin, reversal of susceptibility to this insecticide was significantly faster, confirming the key role of metabolic resistance in the observed resistance. The restoration of susceptibility in a population showing high resistance levels with the use of PBO shows that the incorporation of synergists with the future control strategies may be useful in restoring the effectiveness of the existing tools. All these show that using insecticides of differing modes of action on a selective basis with the addition of synergists for public health/agriculture use, could help to manage resistance in the target populations.

## Conclusions

High intensity of resistance associated with the loss of efficacy of impregnated bed nets was observed in *An*. *gambiae* from Mayuge. This represents a serious threat for vector control. Interestingly, we noticed in the absence of selection pressure a significant reduction in the frequency of Kdr-E together with the expression of key metabolic resistance genes leading to restoration of susceptibility in less than 10 generations supporting that rotation of insecticide can improve vector control against current and importantly for novel insecticides.
